# Contributions for the Medical Volume to Be Published under the Direction of the Sanitary Commission

**Published:** 1866-10

**Authors:** 


Contributions for the Medical Volume to be Published under the direction of the Sanitary Commission.—Our readers are doubtless aware that a series of volumes is to be published under the direction of the Sanitary Commission; the different volumes to be devoted respectively to a History of the Commission, to Hospitals, to Military Hygiene, to Surgery, and to Medicine. We are requested by the editor of the volume on Medicine to invite any who served as surgeons or physicians during the war, either at the North or South, to contribute articles relating to camp diseases. All articles received will be carefully examined, and if incorporated in the work, due acknowledgment will be made to the authors. Articles relating to any of the diseases which prevailed in the armies of the United States or among the Confederate troops, will be gladly received. As the volume will be largely circulated, it will be a desirable medium for the diffusion of important facts and conclusions, based on the experience of those who held medical positions during the war. Articles should be sent, if possible, by the middle of September, or, at the furthest, by the first of October. They may be directed to the Medical Committee of the Sanitary Commission, No. 21 West 12th street, New York City. Editors of Medical Journals are respectfully requested to insert this notice.—*N. Y. Medical Journal.*

